# Genetic polymorphisms in the *ALDH2* gene and the risk of ischemic stroke in a Chinese han population

**DOI:** 10.18632/oncotarget.21803

**Published:** 2017-10-10

**Authors:** Shuaiqi Sun, Jun He, Yao Zhang, Rongjun Xiao, Mengdan Yan, Yulin Ren, Yuanyuan Zhu, Tianbo Jin, Ying Xia

**Affiliations:** ^1^ Department of Neurosurgery, Haikou People's Hospital, Xiangya Medical College Affiliated Haikou Hospital, Central South University, Haikou 570311, Hainan, China; ^2^ Key Laboratory of High Altitude Environment and Genes Related to Diseases of Tibet Autonomous Region, School of Medicine, Xizang Minzu University, Xianyang 712082, Shaanxi, China; ^3^ School of Life Sciences, Northwest University, Xi’an 710069, Shaanxi, China

**Keywords:** ischemic stroke, ALDH2, single-nucleotide polymorphism, case-control study

## Abstract

**Background:**

Previous studies have shown that aldehyde dehydrogenase 2 (*ALDH2*) plays a role in ischemic stroke progression. In recent years, the activation of the *ALDH2* pathway have been reported serving as a useful index in the identification of stroke-prone participants, and the *ALDH2* pathway may be a potential target for the therapeutic intervention in ischemic stroke.

**Materials and Methods:**

We evaluated six tagging single-nucleotide polymorphisms (SNPs) of the *ALDH2* gene in a case–control study from Hainan of China (488 cases, 503 controls). We used SPSS version 17.0 statistical software, Excel software and other analysis software to explore associations between SNPs and the risk of ischemic stroke various genetic models (additive, dominant, and recessive).

**Results:**

Through statistical analysis, we found that *ALDH2* rs886205 [odds ratio (OR) = 6.39; 95% confidence interval (CI) = 1.19-34.38; *p* = 0.03] and rs7296651 (OR = 9.29; 95% CI = 1.37-63.21; *p* = 0.02) were associated with increased risk of ischemic stroke in recessive model analysis. In addition, we established that the “AA” genotype (OR = 5.99; 95% CI = 1.11-32.23; *p* = 0.037) for rs886205 and the “AA” genotype (OR = 8.93; 95% CI = 1.31-60.78; *p* = 0.025) for rs7296651 were associated with increased ischemic stroke risk.

**Conclusions:**

Our results provide evidence that variants of *ALDH2* gene polymorphisms influence the risk of developing ischemic stroke in Han Chinese population.

## INTRODUCTION

Stroke is one important reason of age-related cognitive decline and dementia. As a major cause of adult chronic disease, it is one of the most common causes of death [1, 2]. Stroke is a common higher incidence disease in adult males over the age of 45 which has a strong impact on human health and life. From the data of The World Health Organization, we found that more than 15 million new stroke patients each year which including 5 million fatal or permanent disability cases [3]. More and more studies evidenced genetic predisposition is an important attribution of stroke. However, the identification of genetic molecular mechanisms contributing to stroke is a challenge. Because of ischemic stroke comprises approximately 80% of all stroke cases, and remaining 20% mainly due to primary hemorrhage which induced intracerebral and subarachnoid stroke. The common causes of ischemic stroke include cardiac emboli and small vessel disease that is very critical to research into stroke-susceptibility genes and has taught us attach importance to the classification of patients. In addition, after cardiovascular disease and malignant tumors, acute ischemic stroke has been listed in the third-leading cause of death [4]. The incidence of ischemic stroke could be reduced by control of risk factors, such as hypertension, hypercholesteremia, cigarette smoking, excessive drinking, diabetes mellitus and heart failure. But it only reduces the incidence of ischemic stroke to a certain degree which suggests the existence of other potential risk factors [5, 6].

Identification of novel risk factors which will promote the development of strategies for prevention and treatment of ischemic stroke. From amount of researches, we found various diseases pathophysiological mechanisms, such as atherothrombosis or embolism, can lead to arterial occlusion which is a major cause of stroke. Previous genome-wide association studies (GWAS) identified many genetics variants associated with complex human diseases and have provided unique insights from genetic architecture [7]. Genetic variants associated with risk of ischemic stroke have been revealed by GWAS, these variants often have effects with obvious biological significance [5].

From some studies, we detected the genes encoding the enzymes which metabolize aldehyde dehydrogenase 2 (*ALDH2*) exists polymorphism association with stroke in the pathogenesis process [8, 9]. *ALDH2* gene is located on chromosome 12, which can affect the blood acetaldehyde concentrations. ALDH2 protein is abundantly expressed in normal lung and liver, and is also present in organs that require high mitochondrial content, especially in heart and brain [10]. A previous study has indicated that *ALDH2* gene is able to metabolize some short-chain aliphatic aldehydes, as well as polycyclic aldehydes and so forth [11]. In addition, Several previous studies have assessed SNPs in the *ALDH2* gene for the association with the risk of diseases, such as coronary artery disease [12], diabetes [13], hypertension [14], lacunar infarcts [15]. Recently, a meta-analysis of GWAS has been found a tight association between *ALDH2* genetic variations and pathogenesis of stroke from Asian decedents [16].

Thus, it is possible that locus–locus interactions within the *ALDH2* gene may be associated with risk for ischemic stroke. However, the exact genetic basis of susceptibility to ischemic stroke is still not well defined. To further investigate potential relationships between *ALDH2* polymorphisms and locus–locus interactions in the etiology of ischemic stroke. We randomly selected six tag candidate SNPs (rs886205, rs2238152, rs441, rs4646778, rs671, and rs7296651) from the SNPs in *ALDH2*, and designed the corresponding primers used for each SNP in the present study are listed in Table 1 and performed a case-control association analysis in Han Chinese population. Our study provides sufficient evidence for the association between *ALDH2* gene polymorphisms and the risk of ischemic stroke.

**Table 1:** Primers used for this study

SNP_ID	1st-PCRP	2nd-PCRP	UEP-SEQ
rs886205	ACGTTGGATGTCTCGCTTTTGGGTTTACGG	ACGTTGGATGCCTTTGACCCCAATGTGAAC	GGGCGACCCTGACCT
rs2238152	ACGTTGGATGAATCCCACCTTTATTTAAG	ACGTTGGATGTGTTGTAAAAAGCACCAACC	CCAACCTCAAAGCCAAA
rs441	ACGTTGGATGAGCCTGGGTGCCAGAGAGA	ACGTTGGATGCCCTGACAGCATTCACTTAG	GGTTTTTGTTTGTTTTTTGAG
rs4646778	ACGTTGGATGGTTTTCTGCTATTGGCCCTG	ACGTTGGATGTATGCAGGCAACAAGACAAC	GCAACAAGACAACTGGGAAAT
rs671	ACGTTGGATGCCTTTGGTGGCTACAAGATG	ACGTTGGATGAGGTCCCACACTCACAGTTT	gaTCCCACACTCACAGTTTTCACTT
rs7296651	ACGTTGGATGGGGCAAGACCCAGATTTGAA	ACGTTGGATGCACGTGGCCTGTAACTATGA	gGGCCTGTAACTATGATTTTGATGAA

## RESULTS

**Figure 1:**
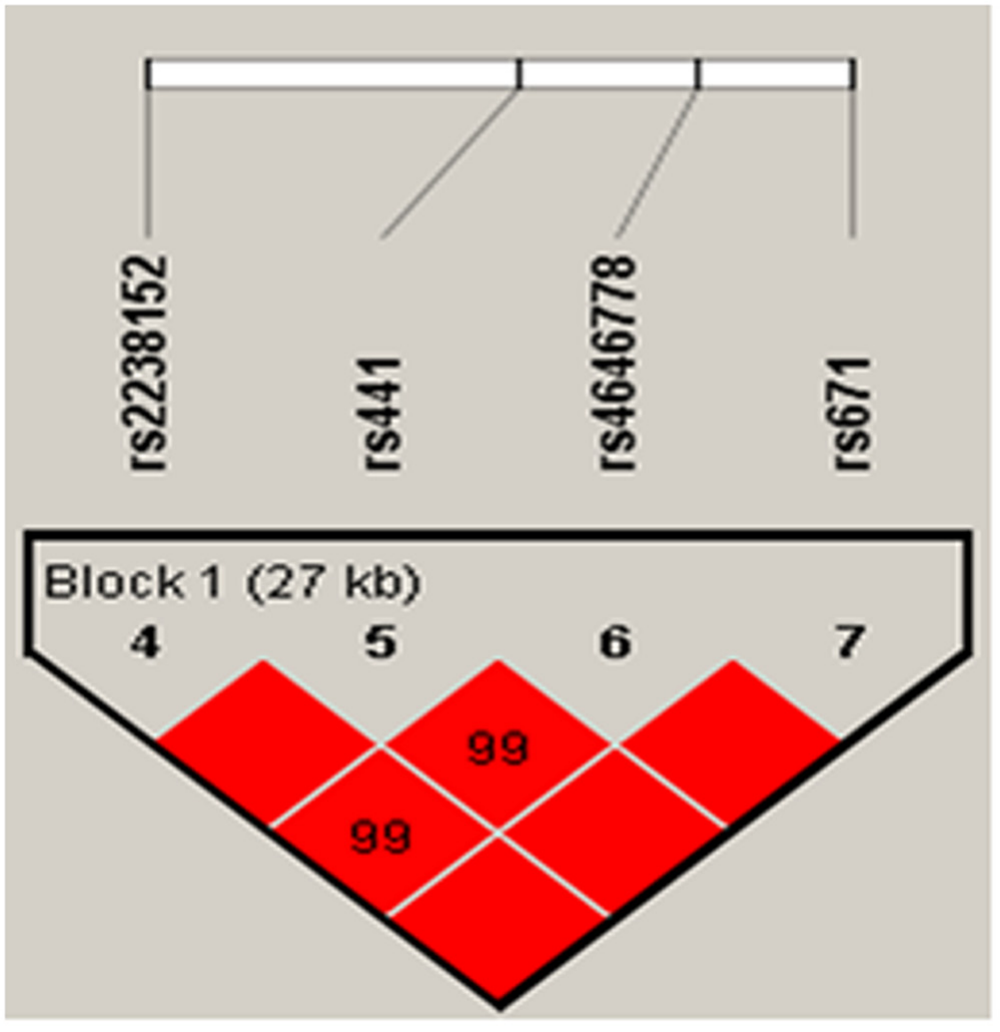
Linkage disequilibrium of polymorphic sites in the *ALDH2* gene

Basic characteristics of all the participants were presented in Table 2. All the tested SNPs are in HWE in the control population of this study (Table 3). Association results between *ALDH2* tSNP genotypes and the risk of stroke were listed in Table 4. We identified two significant SNP genotypes associated with the increased risk of stroke, one was genotype “AA’’ of rs886205 (OR = 5.99; 95% CI = 1.11–32.23; *p* = 0.037) and the other was genotype ‘‘AA’’ of rs7296651 (OR = 8.93; 95% CI = 1.31–60.78; *p* = 0.025). MAF in cases and controls were listed in Table 5. Further model association analyses were performed by logistic tests. Three models – additive, dominant and recessive model – were applied for analyzing the association between polymorphisms and stroke, which was adjusted by the age and gender of the participants. The rs886205 (OR = 6.39; 95% CI = 1.19-34.38; *p* = 0.03) and rs7296651 (OR = 9.29; 95% CI = 1.37-63.21; *p* = 0.02) were observed to be associated with stroke risk in recessive model analyses. By haplotype analysis, one block was explored among the *ALDH2* SNPs (Figure 1). Block 1 contains rs2238152, rs441, rs4646778, and rs671. We performed the association between the block and ischemic stroke, and found the result is statistically insignificant.

**Table 2:** Basic characteristics of controls and cases

Variables	Cases N(%)	Controls N(%)	*p* valve
Age (years)			<0.01^a^
Mean ± SD	63.96 ± 11.06	50.36 ± 7.79	
Sex			<0.01^b^
Male	325 (66.6)	195 (38.8)	
Female	163 (33.4)	308 (61.2)	

^a^ p values were calculated by Student t tests.

^b^ p values were calculated from two-sided Chi-square tests.

**Table 3:** Basic information of candidate SNPs in ALDH2 and associations with stroke

SNP_ID	Chromosome	Position	HWE *p* value	OR (95% CI)	*p* value
rs886205	12q24.12	112204427	0.412	1.08 (0.83-1.40)	0.577
rs2238152	12q24.12	112214459	0.806	1.13 (0.94-1.39)	0.198
rs441	12q24.12	112228849	0.526	0.87 (0.71-1.05)	0.147
rs4646778	12q24.12	112235783	0.614	1.15 (0.95-1.40)	0.151
rs671	12q24.12	112241766	0.814	0.95 (0.74-1.23)	0.712
rs7296651	12q24.12	112246954	0.357	1.04 (0.81-1.34)	0.766

OR, odds ratio; CI, confidence interval; SNP, single nucleotide polymorphism.

**Table 4:** Association between ALDH2 tSNP genotypes and the risk of stroke

SNP_ID	Genotype	No. (frequency)		OR (95% CI)	*p* value
		Case	Control		
rs886205	AA	10 (2.1)	4 (0.8)	5.99 (1.11-32.23)	**0.037**
	AG	106 (21.8)	131 (26)	0.78 (0.53-1.14)	0.192
	GG	371 (76.1)	368 (73.2)	−	
rs2238152	TT	42 (8.6)	41 (8.3)	0.75 (0.41-1.36)	0.34
	TG	210 (43.5)	192 (39)	1.33 (0.94-1.88)	0.109
	GG	231 (47.9)	259 (52.7)	−	
rs441	CC	46 (9.4)	44 (8.7)	0.82 (0.46-1-45)	0.493
	CT	208 (42.6)	192 (38.2)	1.33 (0.95-1.88)	0.1
	TT	234 (48)	267 (53.1)	−	
rs4646778	AA	47 (9.6)	43 (8.6)	0.83 (0.47-1.48)	0.533
	AC	206 (42.2)	192 (38.4)	1.33 (0.94-1.88)	0.103
	CC	234 (48.2)	265 (53)	−	
rs671	AA	11 (2.3)	8 (1.6)	1.21 (0.38-3.90)	0.744
	AG	108 (22.1)	124 (24.7)	0.83 (0.56-1.23)	0.359
	GG	369 (75.6)	371 (73.7)	−	
rs7296651	AA	10 (2.1)	3 (0.7)	8.93 (1.31-60.78)	**0.025**
	AG	118 (24.9)	134 (27)	0.87 (0.60-1.26)	0.449
	GG	346 (73)	359 (72.3)	−	

OR, odd ratio; CI, confidence interval.

**Table 5:** Association of SNPs with risk of stroke based on logistic tests adjusted by gender and age

SNP_ID	Minor allele	MAF (case)	MAF (control)	Additive model		Dominant model		Recessive model	
				OR (95% CI)	*p*	OR (95% CI)	*p*	OR (95% CI)	*p*
rs886205	A	0.129	0.138	0.75 (0.67-1.34)	0.76	0.85 (0.58-1.23)	0.38	6.39 (1.19-34.38)	0.03^*^
rs2238152	T	0.304	0.278	1.03 (0.80-1.32)	0.84	1.20 (0.86-1.66)	0.28	0.66 (0.37-1.17)	0.16
rs441	C	0.307	0.278	1.05 (0.82-1.34)	0.71	1.21 (0.88-1.68)	0.24	0.72 (0.41-1.25)	0.25
rs4646778	A	0.307	0.278	1.05 (0.82-1.35)	0.68	1.22 (0.88-1.28)	0.24	0.74 (0.42-1.28)	0.28
rs671	A	0.133	0.139	0.91 (0.65-1.26)	0.56	0.86 (0.59-1.25)	0.43	1.27 (0.40-4.05)	0.69
rs7296651	C	0.146	0.141	1.04 (0.74-1.46)	0.84	0.94 (0.65-1.35)	0.74	9.29 (1.37-63.21)	0.02^*^

MAF, minor allele frequency; OR, odd ratio; CI, confidence interval.

## DISCUSSION

It is well known that genetic polymorphisms has an effect on the regulation of gene expression, which contributes to the differences between individuals in the susceptibility to a disease and its severity. Stroke is a heterogeneity disease, and the pathogenesis of stroke showed individualization in human. Stroke tends to run in families, it shows that genetic factors affect the development of the stroke. The Framingham study verified parental stroke by 65 years of age resulted in a 2.79-fold independent increase in the risk of offspring stroke [17]. In the present case–control study, we investigated the associations between the 6 SNPs of *ALDH2* and risk of ischemic stroke. To the best of knowledge, we identified rs886205 and rs7296651 in the *ALDH2* gene associated with an increased risk of ischemic stroke. In recent years, one research showed that carrying the wild-type allele of the *ALDH2* polymorphism increased stroke risk among Korean men, but not in Korean women [18]. *ALDH2* may have the same effect on cerebral infarction as on stroke, and cerebral infarction is one of the most common types of ischemic stroke. One previous study in Japanese reported that there was no significant association between the *ALDH2* genotype and the presence of lacunar infarction, but the ALDH2^*^1/^*^1 genotype was significantly associated with the development lacunar infarcts [16]. Besides, Sun et al. [19] found that *ALDH2* can prevent stroke by clearing 4-hydroxy-2-nonenal. In general, these findings indicate that ethnic differences among the *ALDH2* gene variants may affect the development of stroke in different populations.

Stroke is a multifactorial disease attributable to genetic, environmental and other factors. Heavy alcohol consumption has been reported to be a positive risk factor for the susceptibility to stroke. Alcoholism is a complex polygenic behavioral disorder, because of complex gene and environment interactions. Current evidence indicates that ALDH2^*^2 may influence the risk of alcoholism in Koreans [20]. However, the effect of moderate drinking remains controversial. Soo et al. [21] identified that light to moderate alcohol consumption may be associated with a reduced risk of ischemic stroke in a Korean population. Mani et al. [22] suggested that the functional *ALDH2* rs886205 polymorphism does not affect risk for risky alcohol consumption in German populations. This finding is consistent with our experimental results. Therefore, alcohol consumption is not a major factor for stroke, heavy alcohol consumption is just one of the main predisposing factor for stroke.

Heavy alcohol consumption also conferred by *ALDH2* has been reported to be associated with hypertension in men [23]. *ALDH2* is responsible for consumption ethanol in the process of blood pressure. *ALDH2* genetic polymorphism can be altered ethanol pharmacokinetic properties, and leading to accumulation of the acetaldehyde following alcohol consumption. *ALDH2* variants are considered to be governed by the accumulation of acetaldehyde, acetaldehyde is a major metabolic product of ethanol. The mutant ALDH2^*^2 gene was associated with an increased risk of hypertension in human, the blood pressure and *ALDH2* enzymatic activity may be affected by gene and environment, such as life-style and ethnicity. For example, the main symptoms of alcoholism includes the myocardial hypertrophy, derangement of myofibrillary architecture, interstitial fibrosis, which may lead to a greater risk of cardiovascular anomalies such as stroke, and hypertension [24]. Heavy alcohol consumption exhibits a higher systolic blood pressure and a higher overall prevalence of hypertension compared with light to moderate drinkers. It is estimated that one tenth hypertensive cases may be the result of alcohol abuse. The correlation between alcohol intake and blood pressure appears to be independent of any other pathological variables such as diabetes mellitus, age, coronary heart disease, and cigarette smoking. Furthermore, moderate alcohol consumption displays reduced incidence of atherosclerosis, coronary heart diseases, and stroke. Given the hypertension is an important factor of cardiovascular morbidity, limiting alcohol intake is vital prevent measures for patients with hypertension.

Plenty of clinical and experimental studies have indicated that influence of *ALDH2* genotype on blood pressure may be quite complex, depending on the amount of alcohol intake, blood pressure measurement, environmental and genetic factors. Based on these observations, it is plausible that the mutant *ALDH2* serves as a beneficial factor against hypertension by altered drinking behavior and as an independent risk factor for hypertension. Ohsawa et al. [25] reported that *ALDH2* functioned as a protector against oxidative stress and might influence the onset of hypertension and myocardial infarction. They also reported the rs671 GG genotype should be associated with a lower incidence of hypertension. The *ALDH2* SNP rs671 was investigated in alcoholism Mongolians and the results indicated that ALDH2^*^1 genotype allele frequencies were significantly higher in patients with essential hypertension, in conjunction with a much lower frequency of the ALDH2^*^2 alleles in patients with essential hypertension. However, we found no differences in the distribution of the rs761 genetic variants in Han population in our study. These differences may be due to ethnic and regional differences, environmental factors, and dietary habits. In addition, *ALDH2* decreased Ang II-induced ROS generation, thereby preventing ROS-induced constriction. This study describes that *ALDH2* is beneficial in ROS-induced vascular contraction in the AngII hypertensive model. Then *ALDH2* mutation is tied with the increased prevalence of hypertension [26]. However, such increased risk has been associative with alcoholism. Heavy alcohol consumption conferred by *ALDH2* genotype has been reported to be associated with hypertension in men [26]. Considering the hypertension is an important factor of cardiovascular morbidity and stroke is a most important cause of cardiovascular disease, so it is necessary to control blood pressure which would further prevention of cardiovascular disease.

Our study has several advantages. Our population was enrolled from Hainan Province. The region has a high geographic stability, which could significantly reduce the potential confounding effects of the heterogeneous participants in the study. However, some limitations should be considered. First, the sample size (488 cases and 503 controls) of our study was relatively small. Second, correlations between polymorphisms and histological subtypes were not evaluated in this study. Third, we haven't collected the data of vascular risk factors and alcohol drinking behavior to further analysis. Finally, the functions of the genetic variants and the mechanisms have not further explored in this study.

## MATERIALS AND METHODS

### Study population and data collection

For perform the study, we recruited a total of 991 participants (488 cases, 503 controls) from the People's Hospital of Hainan Province during July 2012 to November 2015, and they are all Haikou and the surrounding areas civilians Stroke is divided into ischemic and Hemorrhage. The 488 ischemic stroke patients (325 males, 163 females; mean age 63.93 ± 11.06 years) all met the World Health Organization's stratified criteria for ischemic stroke in our study, and further diagnosed with modern computerized tomography investigative modalities, such as computerized tomographic (CT) and magnetic resonance imaging (MRI). Patients only have a history of the previous stroke, head trauma, brain stroke precipitated during surgery or angiography, excluded with a bleeding diathesis, illicit drug use history, or concomitant serious medical illness such as malignancy, live cirrhosis, sepsis, meningoencephalitis, autoimmune disorders, evidence of cerebral vascular malformation or symptoms of a transient ischaemic attack. None of the patients had suffered any treatment before recruitment. All the patients were all recently diagnosed and histologically identified to be ischemic stroke cases. Meantime, we also recruited a random sample of 503 unrelated healthy individuals as controls (195 males, 308 females; mean age 50.36 ± 7.79 years) from the same hospital, and according to standard recruitment and exclusion criteria. All the participants are Chinese Han ethnic, and were informed the purpose and experimental procedures of the study.

### Demographic and clinical data

A standardized epidemiological questionnaire including age, gender, smoking status, alcohol consumption, residential region, ethnicity, and family history of stroke, was used to collect demographic and personal data. We obtained clinical information for the patients through consulted with their treating physicians or from reviews of their medical charts. After signing an informed consent form, venous blood samples (5 ml) were obtained from each participant. The ethics Committee of the People's Hospital of Hainan Province approved the use of blood samples and the protocol of the research.

### SNP selection and genotyping

Using the NCBI database, we randomly selected six tag candidate SNPs (rs886205, rs2238152, rs441, rs4646778, rs671, and rs7296651) from the SNPs in *ALDH2* gene with minor allele frequencies (MAFs) >5% in the Asian population. Genomic DNA was extracted from the whole blood samples from the 488 cases and the 503 controls using a Blood DNA Extraction Kit (GoldMag Co. Ltd., Xi'an City, China), and DNA concentrations were measured using a NanoDrop 2000 (Thermo Scientific, Waltham, Massachusetts, USA). MassARRAY Assay Design 3.0 Software (Sequenom, San Diego, CA, USA) was used to design the Multiplexed SNP MassEXTEND assay [27], and SNP genotyping was performed using the Sequenom MassARRAY RS1000 (Sequenom Inc., San Diego, CA, USA) system according to the standard protocol. Sequenom Typer 4.0 Software (Sequenom Inc., San Diego, California, USA) was used to manage and analyze the data.

### Statistical analysis

We used SPSS version 17.0 statistical software (SPSS Inc., Chicago, IL, United States) and Excel (Microsoft Corp., Redmond, WA, United States) for statistical analyses. The Chi-squared test and the Student's t-test were used to compare genotype frequencies and allele frequencies between patients and controls. The Hardy–Weinberg equilibrium (HWE) of each SNP was determined by the Chi-squared test, which compared the actual and expected frequencies of the genotypes in the controls. Genetic associations between SNPs and the risk of stroke were tested through various genetic models which including additive, dominant, and recessive models which using SNPStats webpage (http://bioinfo.iconcologia.net). The effects of the polymorphisms on the risk of stroke were reflected as odds ratios (ORs) with 95% confidence intervals (95% CIs) which were calculated by unconditional logistic regression analyses adjusted for age and gender [28]. A linkage disequilibrium (LD) analysis was performed by Haploview v4.2 with genotype data. The pattern of LD was analyzed using D’ and evaluated the haplotypes of the candidate SNPs. All of the statistical tests were two-sided, and *p-*values < 0.05 were statistically significant.

## CONCLUSION

In summary, in this study of a Han Chinese sample, we identified two novel *ALDH2* tSNPs associated with ischemic stroke susceptibility which indicates that *ALDH2* gene may be provide new insights into the etiology of ischemic stroke. To better understand the relationships between gene polymorphisms and stroke progression, additional genetic risk factors and new candidate genes should be identified and analyzed.

### Ethics approval and consent to participate

This research was performed in accordance with the Helsinki Declaration and was approved by the ethics committee of People's Hospital of Hainan Province (Ethics Committee of Research in Humans). Informed consent for this research was conducted under the approval of the ethics committee of People's Hospital of Hainan Province.

## Abbreviations

ALDH2: aldehyde dehydrogenase 2
SNP: single-nucleotide polymorphisms
CI: confidence interval
GWAS: genome-wide association studies
CT: computerized tomographic
MRI: magnetic resonance imaging
HWE: Hardy–Weinberg equilibrium
LD: linkage disequilibrium
